# Investigating the Effects of Pink-Generating Ligands on Enhancing Color Stability and Pigment Properties in Pork Sausage Model Systems Cured with Sodium Nitrite or White Kimchi Powder

**DOI:** 10.3390/foods13182872

**Published:** 2024-09-10

**Authors:** Su Min Bae, Jong Youn Jeong

**Affiliations:** Department of Food Science & Biotechnology, Kyungsung University, Busan 48434, Republic of Korea; smbae219@gmail.com

**Keywords:** nitrite alternative, cured meat color, histidine, cysteine, nicotinamide, white kimchi powder, pork sausage

## Abstract

In this study, we investigated the effects of different nitrite sources (sodium nitrite or white kimchi powder) and pink-generating ligands (cysteine, histidine, or nicotinamide) on the development and stability of cured meat color in pork sausage model systems over 30 d of refrigerated storage. The samples were prepared in a 2 × 3 factorial design with two nitrite sources and three ligands, and their physicochemical properties were evaluated on days 0, 15, and 30. Although white kimchi powder induced cured color development similar to that of synthetic sodium nitrite, it resulted in higher cooking loss and lower residual nitrite content in cured pork sausages (*p* < 0.05). The addition of cysteine resulted in significantly higher CIE a* values, cured meat pigment, and curing efficiency than histidine and nicotinamide (*p* < 0.05), while yielding lower pH values, residual nitrite content, and total pigment content (*p* < 0.05). The storage duration significantly reduced the residual nitrite and total pigment contents of the products. These findings suggest that white kimchi powder can serve as a natural alternative to sodium nitrite in pork sausage models and that the incorporation of cysteine has a favorable impact on the development and enhancement of cured meat color.

## 1. Introduction

The use of nitrite for curing meat has long been practiced in the meat processing industry. It plays an important role in the development of the characteristic pink color of cured meats, suppression of pathogens such as *Clostridium botulinum*, antioxidant effects, and impartation of distinctive flavors [1,2,3]. Traditionally cured meat products have been a key category in the meat processing industry; however, modern consumers have recently shown increased interest in natural meat products [4,5,6]. To accommodate this trend, meat processors and researchers have explored the use of vegetable-based natural substances to replace synthetic nitrites in cured meat [3,7]. Numerous studies have explored natural ingredients such as celery, red beets, Chinese cabbage, radish, Swiss chard, and kimchi [7,8,9,10,11,12,13,14]. These efforts have been valuable in providing alternatives to traditional nitrite use. For example, the incorporation of beetroot powder as a nitrite substitute in Turkish fermented sausages could lead to considerable improvements in product quality, as evidenced by increased redness [12]. A recent study evaluated the potential of radish extracts as natural curing agents for restructured meat products and achieved promising results in terms of color development and sensory acceptance [8].

The source of nitrite can affect the residual nitrite levels in finished products, and products containing plant-based powders generally display a lower residual nitrite content [6,15,16]. Color development of cured meat is a crucial aspect of meat processing, as it may influence consumer perceptions of product quality [15]. However, low levels of residual nitrites can affect the color and pigment stability of the product during storage [17]. Consequently, a solution that can improve overall product quality while addressing this issue is required. 

Fermented vegetable products such as white kimchi have gained increasing attention as potential alternatives to synthetic nitrites. White kimchi, similar to the representative Korean side dish known as *baechu* kimchi (Chinese cabbage kimchi), is a fermented product. Unlike conventional *baechu* kimchi, it does not contain red pepper powder, which is a distinctive feature. White kimchi, which is readily available on the Korean market, has been found to contain high levels of nitrate (1839–2658 mg/kg) [18], and its concentration further increases to 16,778–19,634 mg/kg after being powdered [13,19]. Furthermore, white kimchi powder (WKP) has the potential to serve as a natural source of nitrite, owing to its high nitrate content. In addition, organic acids and antioxidant compounds derived from their ingredients and fermentation by lactic acid bacteria have potential health benefits [13,19,20]. 

The proper selection of ligands is crucial because they can have a significant impact on the physical and chemical properties of meat products, particularly in enhancing color characteristics. Certain pink-generating ligands such as histidine, cysteine, and nicotinamide have been reported to form complexes with heme from myoglobin or hemoglobin in meat and meat products, resulting in improved color stability and pink coloration [21,22,23,24,25,26]. Moreover, it has been established that specific amino acids play crucial roles in meat color development, both independently and in conjunction with nitrite [23,27]. Independently, these amino acids reduce oxidized myoglobin and stabilize it, while in conjunction with nitrite, they enhance the formation and stability of nitrosylmyoglobin, the primary pigment responsible for cured meat color. This dual functionality contributes to the improved color of cured meat products. In relation to this phenomenon, a previous study reported that the addition of L-histidine, either alone or in combination with iron, contributed to the red color of porcine hemoglobin concentrates and maintained a stable a* value during storage [25]. Another study observed the formation of an L-cysteine–hemin complex that exhibited a bright red color in a meat pigment model system [26]. Furthermore, the addition of nicotinamide results in the formation of nicotinamide-denatured globin hemochrome, which contributes to the pink color of meat products [28]. Although some studies have investigated the effects of various ligands on meat and pigment models, limited information is available regarding the use of natural nitrite substitutes and the effects of storage on color development. In particular, the impact of pink-generating ligands cured with different nitrite sources on the color and pigment characteristics of meat products has not yet been clarified and the influence of storage on color stability has not been determined. Thus, it is hypothesized that the use of WKP as a natural substitute for sodium nitrite (SN) will result in similar color development in pork sausage model systems as synthetic SN and that the addition of certain ligands will have a positive effect on the development and enhancement of cured meat color.

Therefore, the aim of this study was to investigate the impact of various nitrite sources (SN and WKP) and ligands (cysteine, histidine, and nicotinamide) on the development of cured meat color in pork sausage model systems over 30 days of refrigeration, specifically focusing on the color stability and pigment properties associated with nitrite.

## 2. Materials and Methods

### 2.1. Chemicals and Preparation of White Kimchi Powder

#### 2.1.1. Materials and Chemicals

Fresh pork ham and back fat were procured from a local processor (Gimhae, Republic of Korea) at 24–48 h postmortem. Chinese cabbages, radishes, and other vegetables were purchased from local markets (Busan, Republic of Korea). Sodium chloride and dextrose were purchased from Fisher Scientific (Loughborough, UK). Sodium tripolyphosphate, sodium nitrite, L-cysteine, and nicotinamide were purchased from Sigma-Aldrich (St. Louis, MO, USA). Sodium ascorbate and L-histidine were purchased from Acros Organics (Geel, Belgium). Bactoferm^®^ CS-300, a starter culture, was purchased from Chr. Hansen (Milwaukee, WI, USA). Opti.Form^®^ powder (Ace S50) was purchased from Corbion NV (Amsterdam, The Netherlands). All other chemicals and solvents utilized in this research were of analytical grade.

#### 2.1.2. Preparation of White Kimchi Powder

White kimchi powder (WKP) was prepared as a nitrite alternative following the method outlined by Choi et al. [19]. Chinese cabbages and radishes were randomly selected as the primary ingredients to produce white kimchi. The vegetables were cleaned, shredded, and cut prior to use. Chinese cabbages were cut in half, and their cut sides were coated with solar salt. They were then soaked in water for 6 h. After rinsing and draining, the cabbages were combined with the shredded radishes, ground garlic, ginger, fermented shrimp, and solar salt. The mixture was left to ferment for two weeks in a refrigerator (K413SS13, LG Electronics, Changwon, Republic of Korea) at 0 °C. Fermented white kimchi was blended, vacuum-packed in nylon/polyethylene bags using a vacuum packaging machine (DP-901, Dew Pack Machinery, Yongin, Republic of Korea), and stored at −80 °C. It was then dried at −40 °C in a vacuum freeze dryer (PVTFD10R, Ilshinbiobase, Yangju, Republic of Korea) for 2 d and pulverized with a blender. The resulting powder, screened with a 30-mesh sieve, was vacuum-packed and stored at −18 °C until use. Before processing the cured pork sausages, the nitrate concentration of WKP was analyzed using the zinc reduction method described by Merino [29] and was further standardized to approximately 20,000 ppm by adding maltodextrin (#186785579, ES food, Gunpo, Republic of Korea).

### 2.2. Preparation of Cured Pork Sausages Model

The experimental design was a 2 × 3 factorial design with two nitrite sources (50 ppm SN or 0.25% WKP) and three ligands (0.5% cysteine, histidine, or nicotinamide). Purchased pork hams were ground using a grinder (3 mm plate; TC-22 Elegnant plus, Tre Spade, Torino, Italy) along with the back fat after removing visible connective tissues. The ground materials were randomly divided into six portions to manufacture cured pork sausage models (Table 1). SN-C, SN-H, and SN-N samples used SN as the nitrite source and cysteine, histidine, and nicotinamide as the pink-promoting ligands, respectively. However, in the WKP-C, WKP-H, and WKP-N treatments, WKP was added instead of SN as a nitrite source, and a starter culture was added to convert the nitrate contained in the powder into nitrite. These treatments also incorporated cysteine, histidine, or nicotinamide as pink-promoting ligands. As shown in Table 1, each sample was processed according to its formulation ratio. Briefly, ground pork ham and back fat were mixed in a food processer (5K5SS, Whirlpool, St. Joseph, MI, USA) along with sodium chloride and sodium tripolyphosphate for 5 min. Other ingredients, including the nitrite sources and pink-generating ligands, were added along with ice/water and mixed for 7 min. The resulting mixtures were stuffed sequentially into 24 mm diameter NOJAX^®^ cellulose casings (Viskase^®^ Companies, Lombard, IL, USA). The filled samples (SN-C, SN-H, and SN-N) were placed at 3 °C for 2 h for traditional curing, whereas the WKP-C, WKP-H, and WKP-N samples were maintained at 40 °C for 2 h for alternative curing. After curing, the samples (45 sausages from each batch) were placed in a water bath at 90 °C and cooked until the core temperature reached 75 °C. After cooking, all samples were cooled on slurry ice for 20 min, and the sausages from each batch were individually placed in polyethylene pouches, vacuum-packed, and stored at 3 °C in the dark for 30 days. In this study, the pork sausages were individually processed thrice on different days. All experimental parameters, excluding cooking loss, were measured and analyzed periodically on days 0, 15, and 30.

### 2.3. Methods and Analyses

#### 2.3.1. Cooking Loss, Purge Loss, and pH Determination

To determine cooking loss, the sausages were weighed before and after cooking and the drips were removed using a paper towel. Cooking loss was calculated as the percentage of weight difference before and after cooking. For purge loss, the cooked sausages were weighed on processing day (day 0) and storage days (days 15 and 30). After removing the casings and drips, the percentage weight difference before and after storage was calculated. Five sausages/treatments per replicate were used to measure the cooking and purge losses. The pH was measured by adding 90 mL of distilled water to 10 g of cooked sausage, which was then homogenized, and the measurement was performed using an Accumet^®^ AB150 pH meter (Thermo Fisher Scientific, Singapore) following calibration. Two sausages were used per treatment in each replicate, and the pH of each sample was measured in duplicate.

#### 2.3.2. Instrumental Color Measurement

The instrumental color was measured using a Minolta CR-400 colorimeter (Konica Minolta, Osaka, Japan) based on the CIE L*a*b* system. Before measurement, the equipment was set to an 8 mm aperture, illuminant C, and a 2° observer angle and calibrated using a white tile. CIE color measurements were taken on each sausage sample by cutting them parallel to the longitudinal direction. Immediately after cutting, two random measurements were performed on each side to prevent color fading [30]. Four readings were recorded for two sausages per treatment in each replicate. 

#### 2.3.3. Residual Nitrite Analysis

The concentration of residual nitrite in the cured sausages was assessed using the colorimetric AOAC method 973.31 [31]. The analysis was conducted by preparing a standard curve with SN solution, and the results were reported in parts per million (ppm). Two sausage samples per treatment were used for each replicate.

#### 2.3.4. Determination of Cured Meat Pigment, Total Pigment, and Curing Efficiency

The cured meat pigment and total pigment contents were analyzed according to the method described by Hornsey [32]. Briefly, samples were blended with 80% acetone and acidified acetone solution to measure nitrosyl hemochrome and total pigment contents, respectively. The absorbance of the filtrate was measured at 540 and 640 nm using a spectrophotometer (UV-1800, Shimadzu, Kyoto, Japan). The results are reported in ppm. Two sausage samples per treatment per replicate were used to measure the cured meat pigment and total pigment. The curing efficiency was calculated as a percentage of the proportion of cured pigment to total pigment [30].

### 2.4. Statistical Analysis

All experiments were conducted in triplicates. As described in Section 2.2, the experimental model had a 2 × 3 factorial design. Two nitrite sources (50 ppm SN or 0.25% WKP) and three ligands (0.5% cysteine, histidine, or nicotinamide) were assigned to the split plots. Storage duration (days 0, 15, and 30) was treated as a subplot within each treatment. The statistical analysis of the main factors and their interactions was performed using the SAS PROC GLIMMIX procedure (SAS Institute 2012). The least-squares mean was obtained using the LINES option in the SAS PROC GLIMMIX procedure when statistical significance was found at *p* < 0.05.

## 3. Results and Discussion

### 3.1. Effects of Nitrite Sources, Ligands, and Storage Duration on the Physicochemical Characteristics of Cured Pork Sausage Model Systems

#### 3.1.1. Main Factors and Their Interactions

The three main factors of nitrite sources, ligands, and storage duration and their combined effects on the physicochemical properties investigated in this study are shown in Table 2. Two-way interactions were only found between nitrite sources and ligands on CIE L* values and total pigment (*p* < 0.05), and between ligands and storage duration on pH and total pigment (*p* < 0.05). However, no three-way interactions were found between the three main factors for any of the dependent variables (*p* > 0.05).

#### 3.1.2. Cooking Loss, Purge Loss, and pH Values

Pork sausages cured using WKP as the nitrite source exhibited a higher cooking loss (*p* < 0.05) than those cured with SN (Table 3). The results regarding cooking loss in meat products cured with different nitrite sources have varied across studies. A previous study reported no difference in cooking loss between products containing nitrite and naturally cured products [10], whereas another study found that products containing vegetable alternatives resulted in a higher cooking loss [13], which is in accordance with our findings. This phenomenon is attributed to the organic acids present in vegetable alternatives such as WKP [33,34]. In this study, the ligands did not affect cooking loss (*p* > 0.05; Table 2). The addition of cysteine, histidine, and nicotinamide as pink-generating ligands resulted in similar cooking losses (*p* > 0.05; Table 3), suggesting that the ligands did not affect the water retention capacity of the cooked products.

The nitrite sources had no effect on the purge loss of cured pork sausages (*p* > 0.05; Table 2). However, purge loss in cured pork sausages was highly significant with the addition of ligands (*p* < 0.0001; Table 2), with cysteine treatment showing higher purge loss than histidine and nicotinamide treatments (*p* < 0.05; Table 3). This observed difference may be attributed to the differences in their pH levels [35]. Furthermore, as expected, purge loss increased over the storage duration (*p* < 0.05; Table 2 and Table 3), but at a low level of less than 2%. 

The pH values were significantly influenced by nitrite sources (*p* < 0.05), ligands (*p* < 0.0001), and storage duration (*p* < 0.05), as shown in Table 2. The pH of cured pork sausages prepared with WKP was higher than that of sausages prepared with added nitrite (*p* < 0.05); however, the difference in the numerical values was minimal (Table 3). Sucu and Turp [12] replaced synthetic nitrite with beetroot powder in fermented beef sausages and found that the pH values were the same as those of their counterparts, contradicting the results of the present study. Nonetheless, Jeong et al. [9] demonstrated that the incorporation of Chinese cabbage powder resulted in pH values that were higher than those of the nitrite-added control, which aligns with the findings of the current study. Regarding the influence of added ligands, the lowest and highest pH values were observed when cysteine and histidine were added, respectively (*p* < 0.05; Table 3). Cysteine is generally categorized as a neutral amino acid; however, its side-chain sulfhydryl group enables it to behave as a weak acid by donating hydrogen ions [36]. Histidine is commonly recognized as a basic amino acid [37]. Thus, the outcome of the cured pork sausage model seems to be influenced by amino acid characteristics. Additionally, the pH values of the cured pork sausages remained constant until day 15 but showed a significant decrease by day 30 (*p* < 0.05; Table 3). Although significant, the numerical values were small between the storage durations.

#### 3.1.3. Instrumental Color

The CIE L* values of the cured pork sausages were influenced by the nitrite sources (*p* < 0.0001) and ligands (*p* < 0.0001) but not by the storage duration (*p* > 0.05) (Table 2). Cured pork sausages treated with WKP exhibited lower CIE L* values than those treated with SN (*p* < 0.05; Table 3). Jeong et al. [9] posited that the color of naturally cured meat products is influenced by the color of plant-based alternatives, specifically noting that powders derived from leafy vegetables yield lower CIE L* values for the finished products. The addition of nicotinamide to cured pork sausages resulted in the highest CIE L* values (*p* < 0.05; Table 3), followed by significantly lower CIE L* values for the addition of cysteine and histidine (*p* < 0.05). However, storage for 30 d did not affect the CIE L* values of cured pork sausages (*p* > 0.05; Table 3).

In cured meat products, redness is widely regarded as an indicator of characteristic pigmentation (nitrosyl hemochrome) [2,30]. In this study, the results showed that nitrite sources (*p* < 0.05) and ligands (*p* < 0.05) had a significant impact on the CIE a* values of cured pork sausages, whereas storage duration (*p* > 0.05) did not have a significant effect (Table 2). The use of WKP in naturally cured pork sausages resulted in higher CIE a* values than those cured with SN (*p* < 0.05; Table 3). These results differ from those of previous studies [14,38], which reported no significant difference in CIE a* values between naturally and conventionally cured products. However, Sucu and Turp [12] reported that beef sausages containing beetroot powder had higher CIE a* values than nitrite-containing products, which supports the findings of this study. However, the color of the vegetable-based alternative may have affected the color of the final product, and the addition of WKP could positively affect the development of redness. Among the added ligands, the addition of cysteine resulted in an increase in the CIE a* values of the cured pork sausages compared with the addition of histidine or nicotinamide (*p* < 0.05; Table 3). However, there was no difference in the CIE a* values between histidine and nicotinamide treatments (*p* > 0.05). It has been reported that cured meat products with a lower pH exhibit higher redness because acidic conditions can accelerate the curing reaction of nitrite conversion to nitric oxide [39], and a similar trend was observed in the current study. However, the CIE a* values did not change regardless of storage duration (*p* > 0.05; Table 3).

Nitrite sources had a significant effect on the CIE b* values of cured pork sausages (*p* < 0.05; Table 2). Pork sausages treated with WKP exhibited higher CIE b* values than those treated with SN (*p* < 0.05; Table 3). Some studies have reported that the use of plant sources as substitutes for SN leads to an increase in the CIE b* values of pork sausages and ham [40,41]. Consequently, the differences in color observed in alternatively cured meat products are often attributed to plant sources [40]. In the current study, it was speculated that the increase in yellowness of pork sausages processed with white kimchi was due to the influence of chlorophyll and carotenoids present in Chinese cabbage, which is a primary ingredient in white kimchi [42]. Although no ligand effects were detected on the CIE b* values (*p* > 0.05; Table 2 and Table 3), storage duration significantly influenced the CIE b* values of cured pork sausages (*p* < 0.05; Table 2). Specifically, the CIE b* values increased after day 15 of storage compared to day 0 (*p* < 0.05; Table 3). The increase in the CIE b* value in sausages during storage may be due to oxidative processes, particularly lipid oxidation and pigment degradation [43,44].

#### 3.1.4. Residual Nitrite Content

**Figure 1:**
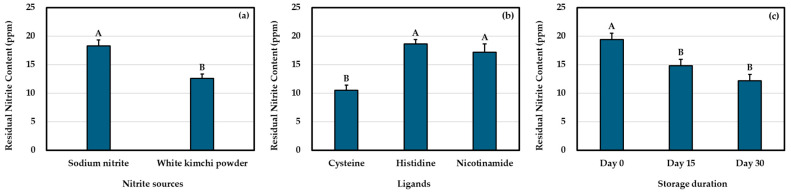
Effects of (**a**) nitrite sources, (**b**) ligands, and (**c**) storage duration on residual nitrite content in cured meat model systems. Different letters within each main factor indicate significant differences (*p* < 0.05).

The residual nitrite content in cured pork sausages can vary depending on the level of nitrite added, type of product, processing conditions, presence of reducing agents, pH, storage conditions, and correlation with other additives [12,39,45]. In the present study, the residual nitrite content of pork sausages was influenced by all three factors (*p* < 0.05; Table 2). The addition of WKP as a natural curing agent resulted in a lower residual nitrite content (12.62 ppm) than that of the traditionally cured products (18.30 ppm) (*p* < 0.05; Figure 1a). These results are consistent with those of previous reports on naturally cured meat products, showing a tendency towards a lower residual nitrite content than that of traditionally cured products [11,13,15]. This difference is attributed to the presence of organic compounds, such as vegetable-based alternatives, which contain bioactive compounds [11,12,33] that are believed to accelerate the loss of nitrite. Moreover, the addition of cysteine to cured pork sausages resulted in a significantly lower residual nitrite content (10.53 ppm) than the addition of histidine (18.66 ppm) or nicotinamide (17.19 ppm) (*p* < 0.05; Figure 1b). However, the residual nitrite content of cured pork sausages treated with histidine and nicotinamide was similar (*p* > 0.05). The reduction in residual nitrite by the addition of cysteine appears to be due to its sulfhydryl group. Furthermore, the ability of cysteine to function as a reducing agent is facilitated by the presence of a sulfhydryl group, which results in an increase in its reducing capacity and a concomitant decrease in residual nitrite content [23,24,46]. It is well known that nitrite levels decrease in cured meat products as the storage duration increases [17,47,48]. In the current study, the residual nitrite in cured pork sausages decreased from 19.41 to 14.80 ppm during storage from days 0 to 15 (*p* < 0.05; Figure 1c). However, there was no change in the residual nitrite content during storage from days 15 to 30 (*p* > 0.05).

#### 3.1.5. Cured Meat Pigment, Total Pigment, and Curing Efficiency

The nitrite sources did not have a significant effect on the cured meat pigment (nitrosyl hemochrome) in cured pork sausages (*p* > 0.05; Table 2 and Table 4). Studies on meat products using natural alternatives to SN have generally found low residual nitrite levels; however, the cured meat pigment was similar to that of its counterparts [11,13]. Natural sources, such as WKP, used in alternative curing methods, are composed of nitrate, nitrite, and other components [33,45]. These components are believed to aid the formation of nitric oxide from nitrite and promote sequential nitric oxide and myoglobin reactions within meat [11]. The results of the current study demonstrated these effects. The ligand effect on cured meat pigment in cured pork sausages was significant (*p* < 0.05; Table 2). Notably, the addition of cysteine to cured pork sausages resulted in a higher cured meat pigment compared to samples treated with histidine or nicotinamide (*p* < 0.05; Table 4). However, the addition of histidine and nicotinamide did not affect the cured meat pigment in cured pork sausages (*p* > 0.05). Reith and Szakaly [49] suggested that cysteine promoted the formation of nitric oxide myoglobin. The highly cured meat pigment observed in the current study appears to be the result of the reducing ability of cysteine [23,25]. However, storage duration did not affect the cured meat pigment in cured pork sausages (*p* > 0.05, Table 2 and Table 4), and the cured meat pigment remained stable until the end of the storage period (day 30).

The total pigment in cured pork sausages was not affected by the nitrite sources (*p* > 0.05; Table 2 and Table 4) but was significantly affected by the ligands (*p* < 0.0001) and storage duration (*p* < 0.0001). Histidine and nicotinamide resulted in a significantly higher total pigment content in cured pork sausages compared to cysteine (*p* < 0.05; Table 4). Although the exact mechanism underlying the difference in pigment content due to these ligands is not well understood, it is possible that the reduction in total pigment content in sausages containing cysteine is attributable to the higher purge loss observed during storage. Furthermore, the total pigment content in the cured pork sausages decreased over time, with the total pigment on day 30 being lower than that on days 0 and 15 (*p* < 0.05; Table 4).

Curing efficiency is typically expressed as a percentage of the conversion of nitroso pigment to total pigment, with a higher efficiency indicating more effective meat curing (King et al., 2023). In the current study, nitrite sources did not affect the curing efficiency of cured pork sausages (*p* > 0.05, Table 2 and Table 4). Recent studies using natural ingredients to replace synthetic nitrite have reported curing efficiency, which is consistent with the results of the current study [8,50]. This is probably due to a similar curing reaction, with only the source of nitrite being different [3]. Nevertheless, the curing efficiency of cured pork sausages was affected by the added ligands (*p* < 0.0001; Table 2). Cysteine increased the curing efficiency of cured pork sausages compared to those containing histidine or nicotinamide (*p* < 0.05; Table 4); however, neither histidine nor nicotinamide resulted in a difference in curing efficiency (*p* > 0.05). According to Hornsey [51], the proportion of nitroso pigment to total pigment in cooked cured pork is inversely proportional to the pH, whereas it increases with cysteine content. Thus, the results of the current study support the finding that cured pork sausages containing cysteine show a higher curing efficiency, whereas the addition of histidine or nicotinamide results in a relatively lower curing efficiency. However, storage duration did not affect the curing efficiency of cured pork sausages (*p* > 0.05; Table 2 and Table 4).

### 3.2. Combined Effects of Nitrite Sources and Ligands or Ligands and Storage Duration on CIE L*, pH, and Total Pigment of Cured Pork Sausage Model Systems

#### 3.2.1. Combined Effects of Nitrite Sources and Ligands on CIE L* and Total Pigment

The CIE L* value of pork sausages cured with WKP was reduced by the addition of cysteine or histidine compared with those cured with SN (*p* < 0.05; Table 5). However, the addition of nicotinamide did not result in a difference in the CIE L* values of pork sausages cured using either of the two nitrite sources (*p* > 0.05). In contrast, for pork sausages cured with SN or WKP, the addition of nicotinamide increased the CIE L* value the most (*p* < 0.05), whereas histidine decreased the CIE L* value (*p* < 0.05).

The total pigment of pork sausages cured with either of the two nitrite sources was not affected by the ligands, except for cysteine treatment (*p* > 0.05; Table 5). The addition of cysteine resulted in a decrease in the total pigment of pork sausages treated with either SN or WKP, whereas the effects of histidine and nicotinamide on the total pigment were not significant (*p* > 0.05).

#### 3.2.2. Interaction Effects of Ligands and Storage Duration on pH and Total Pigment

The pH of the cured pork sausages with cysteine or histidine remained stable over 30 d of storage (*p* > 0.05; Table 6), whereas the pH of the products cured with nicotinamide decreased after 15 d of storage (*p* < 0.05). For each storage period, pork sausages cured with cysteine had lower pH values than those cured with histidine or nicotinamide, whereas those cured with histidine maintained the highest pH levels, except on day 0 (*p* < 0.05).

Cured pork sausages treated with cysteine exhibited a decrease in total pigment on day 30 compared with that on days 0 and 15 (*p* < 0.05; Table 6). However, histidine addition did not change the total pigment of the cured pork sausages over the entire storage period (*p* > 0.05). Cured pork sausages with nicotinamide showed no difference in total pigment between days 0 and 15 (*p* > 0.05) but decreased on day 30 compared to day 15 (*p* < 0.05). The total pigment content of cured pork sausages at each storage period was similar between the cysteine and nicotinamide treatments, except on day 0, when the cysteine treatments had lower contents than the histidine and nicotinamide treatments (*p* < 0.05). However, there was no difference in the total pigment content between histidine and nicotinamide treatments at any storage period (*p* > 0.05).

## 4. Conclusions

Although cured pork sausages processed with WKP exhibited low residual nitrite levels, they possessed cured meat pigment characteristics similar to those of sausages treated with SN. Among the pink-generating ligands tested, the addition of cysteine had a positive effect on the development and enhancement of cured meat color of pork sausages, regardless of the nitrite sources. The storage duration reduced the residual nitrite and total pigment contents of the cured pork sausages. Therefore, WKP can serve as a potential natural substitute for synthetic nitrite, and proper selection of ligands is crucial for enhancing the color characteristics of meat products. Future research should explore the potential applications of other natural nitrite sources and ligands in cured meat production, while also examining their effects on consumer sensory attributes and microbiological safety.

## Figures and Tables

**Table 1:** Formulation for pork sausage models cured with sodium nitrite or white kimchi powder along and different pink-generating ligands.

Ingredients (%)	Sample Code ^1^					
	SN-C	SN-H	SN-N	WKP-C	WKP-H	WKP-N
Pork ham	70.000	70.000	70.000	70.000	70.000	70.000
Pork backfat	15.000	15.000	15.000	15.000	15.000	15.000
Ice/water	15.000	15.000	15.000	15.000	15.000	15.000
Subtotal	100.000	100.000	100.000	100.000	100.000	100.000
Sodium chloride	1.500	1.500	1.500	1.500	1.500	1.500
Sodium tripolyphosphate	0.300	0.300	0.300	0.300	0.300	0.300
Dextrose	1.000	1.000	1.000	1.000	1.000	1.000
Sodium ascorbate	0.050	0.050	0.050	0.050	0.050	0.050
Opti.Form powder	0.500	0.500	0.500	0.500	0.500	0.500
Sodium nitrite (SN)	0.005	0.005	0.005	–	–	–
White kimchi powder (WKP)	–	–	–	0.250	0.250	0.250
Starter culture	–	–	–	0.025	0.025	0.025
Cysteine	0.500	–	–	0.500	–	–
Histidine	–	0.500	–	–	0.500	–
Nicotinamide	–	–	0.500	–	–	0.500

^1^ Sample codes: SN-C, 0.005% SN + 0.5% cysteine; SN-H, 0.005% SN + 0.5% histidine; SN-N, 0.005% SN + 0.5% nicotinamide; WKP-C, 0.25% WKP + 0.5% cysteine; WKP-H, 0.25% WKP + 0.5% histidine; WKP-N, 0.25% WKP + 0.5% nicotinamide.

**Table 2:** Significance and combined effects of nitrite sources, ligands, and storage duration on the quality properties of cured pork sausage model systems.

Main Factors and Their Interactions	Cooking Loss	Purge Loss	pH	CIE L*	CIE a*	CIE b*	Residual Nitrite Content	Cured Meat Pigment	Total Pigment	Curing Efficiency
Nitrite sources ^1^ (N)	*	NS	*	**	*	*	*	NS	NS	NS
Ligands ^2^ (L)	NS	**	**	**	*	NS	*	*	**	**
Storage duration ^3^ (S)	–	*	*	NS	NS	*	*	NS	**	NS
N × L	NS	NS	NS	*	NS	NS	NS	NS	*	NS
N × S	–	NS	NS	NS	NS	NS	NS	NS	NS	NS
L × S	–	NS	*	NS	NS	NS	NS	NS	*	NS
N × L × S	–	NS	NS	NS	NS	NS	NS	NS	NS	NS

Main factors and their combined effects: * = *p* < 0.05, ** = *p* < 0.0001, ‘NS’ denotes no significant difference (*p* > 0.05), and ‘–’ denotes not measured. ^1^ Ground pork meat mixtures were cured with SN or WKP. ^2^ The meat mixtures for curing from different nitrite sources were mixed with cysteine, histidine, or nicotinamide and cooked after curing was completed. ^3^ All cooked sausages were stored at 3 °C in the dark and analyzed on days 0, 15, and 30 of storage.

**Table 3:** Effects of nitrite sources, ligands, and storage duration on cooking loss, purge loss, pH, and CIE color in cured pork sausage model systems.

Main Factors	Cooking Loss (%)	Purge Loss (%)	pH	CIE L*	CIE a*	CIE b*
Nitrite sources ^1^						
SN	1.13 ^B^	1.34 ^A^	6.26 ^B^	67.61 ^A^	9.77 ^B^	6.22 ^B^
WKP	1.45 ^A^	1.28 ^A^	6.31 ^A^	66.73 ^B^	10.34 ^A^	6.41 ^A^
S.E.	0.04	0.14	0.01	0.24	0.15	0.14
Ligands ^2^						
Cysteine	1.34 ^A^	1.86 ^A^	6.15 ^C^	67.21 ^B^	10.80 ^A^	6.24 ^A^
Histidine	1.33 ^A^	1.11 ^B^	6.40 ^A^	66.21 ^C^	9.86 ^B^	6.24 ^A^
Nicotinamide	1.20 ^A^	0.97 ^B^	6.30 ^B^	68.10 ^A^	9.50 ^B^	6.46 ^A^
S.E.	0.05	0.15	0.01	0.25	0.18	0.15
Storage duration ^3^						
Day 0	–	–	6.30 ^A^	67.14 ^A^	9.82 ^A^	6.13 ^B^
Day 15	–	1.04 ^B^	6.29 ^A^	67.29 ^A^	10.25 ^A^	6.39 ^A^
Day 30	–	1.58 ^A^	6.26 ^B^	67.08 ^A^	10.09 ^A^	6.42 ^A^
S.E.	–	0.14	0.01	0.25	0.18	0.15

^A–C^ indicate that superscript letters within a column are significantly different (*p* < 0.05). ‘–’ denotes not measured and S.E. indicates standard error of the mean. ^1^ Ground pork meat mixtures were cured with SN or WKP. ^2^ The meat mixtures for curing from different nitrite sources were mixed with cysteine, histidine, or nicotinamide and cooked after curing was completed. ^3^ All cooked sausages were stored at 3 °C in the dark and analyzed on days 0, 15, and 30 of storage.

**Table 4:** Effects of nitrite sources, ligands, and storage duration on cured meat pigment, total pigment, and curing efficiency in cured meat model systems.

Main Factors	Cured Meat Pigment (ppm)	Total Pigment (ppm)	Curing Efficiency (%)
Nitrite sources ^1^			
SN	28.64 ^A^	42.75 ^A^	67.39 ^A^
WKP	30.50 ^A^	42.67 ^A^	71.68 ^A^
S.E.	0.68	2.10	3.38
Ligands ^2^			
Cysteine	33.29 ^A^	41.68 ^B^	79.94 ^A^
Histidine	27.31 ^B^	43.32 ^A^	63.25 ^B^
Nicotinamide	28.11 ^B^	43.12 ^A^	65.41 ^B^
S.E.	0.83	2.10	3.56
Storage duration ^3^			
Day 0	28.95 ^A^	42.93 ^A^	67.71 ^A^
Day 15	29.46 ^A^	43.21 ^A^	68.38 ^A^
Day 30	30.29 ^A^	41.99 ^B^	72.52 ^A^
S.E.	0.83	2.10	3.56

^A,B^ indicate that superscript letters within a column are significantly different (*p* < 0.05). S.E. indicates the standard error of the mean. ^1^ Ground pork meat mixtures were cured with SN or WKP. ^2^ The meat mixtures for curing from different nitrite sources were mixed with cysteine, histidine, or nicotinamide and cooked after curing was completed. ^3^ All cooked sausages were stored at 3 °C in the dark and analyzed on days 0, 15, and 30 of storage.

**Table 5:** Combined effects of nitrite sources ^1^ and ligands ^2^ on CIE L* and total pigment in cured meat model systems.

Combined Effects	CIE L*			Total Pigment (ppm)		
	Cysteine	Histidine	Nicotinamide	Cysteine	Histidine	Nicotinamide
SN	67.55 ^Ay^	67.01 ^Az^	68.27 ^Ax^	42.27 ^Ay^	43.12 ^Ax^	42.84 ^Axy^
WKP	66.86 ^By^	65.41 ^Bz^	67.92 ^Ax^	41.08 ^By^	43.52 ^Ax^	43.41 ^Ax^

^A,B^ indicate that superscript letters within a column are significantly different (*p* < 0.05; standard error of CIE L*, 0.2769; standard error of total pigment, 2.1043). ^x–z^ denotes that the superscript letters within a row are significantly different (*p* < 0.05; standard error of CIE L*, 0.2769; standard error of total pigment, 2.1043). S.E. indicates standard error of the mean. ^1^ Ground pork meat mixtures were cured with SN or WKP. ^2^ The meat mixtures for curing from different nitrite sources were mixed with cysteine, histidine, or nicotinamide and cooked after curing was completed.

**Table 6:** Combined effects of ligands ^1^ and storage duration ^2^ on pH and total pigment in cured meat model systems.

Combined Effects	pH			Total Pigment (ppm)		
	Cysteine	Histidine	Nicotinamide	Cysteine	Histidine	Nicotinamide
Day 0	6.15 ^Ay^	6.39 ^Ax^	6.35 ^Ax^	42.50 ^Ay^	43.27 ^Ax^	43.01 ^ABxy^
Day 15	6.17 ^Az^	6.43 ^Ax^	6.29 ^By^	42.42 ^Ay^	43.52 ^Ax^	43.69 ^Ax^
Day 30	6.14 ^Az^	6.39 ^Ax^	6.26 ^By^	40.12 ^By^	43.18 ^Ax^	42.67 ^Bx^

^A,B^ indicate that superscript letters within a column are significantly different (*p* < 0.05; standard error of pH, 0.0185; standard error of total pigment, 2.1089). ^x–z^ denotes that the superscript letters within a row are significantly different (*p* < 0.05; standard error of pH, 0.0185; standard error of total pigment, 2.1089). ^1^ Ground pork meat mixtures were cured with SN or WKP, mixed with cysteine, histidine, or nicotinamide, and cooked after curing was completed. ^2^ All cooked sausages were stored at 3 °C in the dark and analyzed on days 0, 15, and 30 of storage.

## Data Availability

The original contributions presented in the study are included in the article; further inquiries can be directed to the corresponding author.
